# Effects of different fertilization rates on growth, yield, quality and partial factor productivity of tomato under non-pressure gravity irrigation

**DOI:** 10.1371/journal.pone.0247578

**Published:** 2021-03-12

**Authors:** Qing-Jie Du, Huai-Juan Xiao, Juan-Qi Li, Jia-Xin Zhang, Lu-Yao Zhou, Ji-Qing Wang

**Affiliations:** College of Horticulture, Henan Agricultural University, Zhengzhou, Henan, China; Northwest Agriculture and Forestry University, CHINA

## Abstract

To select the optimum fertilizer application under specific irrigation levels and to provide a reliable fertigation system for tomato plants, an experiment was conducted by using a microporous membrane for water-fertilizer integration under non-pressure gravity. A compound fertilizer (N:P_2_O_5_:K_2_O, 18:7:20) was adopted for topdressing at four levels, 1290 kg/ha, 1140 kg/ha, 990 kg/ha, and 840 kg/ha, and the locally recommended level of 1875 kg/ha was used as the control to explore the effects of different fertilizer application rates on growth, nutrient distribution, quality, yield, and partial factor of productivity (PFP) in tomato. The new regime of microporous membrane water-fertilizer integration under non-pressure gravity irrigation reduced the fertilizer application rate while promoting plant growth in the early and intermediate stages. Except for the 990 kg/ha fertilizer treatment, yields per plant and per plot for each fertilizer application rate were higher than or equal to those of the control. The new regime could effectively improve PFP and reduce soil nutrient enrichment. Fertilizer at 840 kg/ha showed the optimum results by increasing PFP by 75.72% as compared to control. In conclusion, the fertilizer rate at 840 kg/ha has not only maintained the productivity of soil but also tomato growth and quality of fruit which makes the non-pressure gravity irrigation a potential and cost-effective way for fertilizer application.

## Introduction

Fertilizers, which are indispensable and the most important material input in modern agricultural production [1, 2], have played a vital role in improving the yield and quality of crops [3–5]. However, over-fertilization not only inhibits the improvement of crop yield and quality, it also results in serious issues, such as hardening and acidification of the soil, aggravation of crop pests, leaching loss of soil nutrients, and threats to groundwater safety. These issues exert a serious impact on agricultural sustainability and the ecological environment [6–8]. In recent years, integrated techniques of water and fertilizer application have been widely developed and popularized. These studies have conducted irrigation and fertilization trials according to the soil nutrient status and the crop water and fertilizer requirements, thus achieving the purpose of saving water and fertilizer, increasing production and quality, and protecting the ecological environment [9–11]. The microporous membrane water-fertilizer integration technique is newly developed. By adopting microporous membranes as a substitute for drip irrigation tapes and pipes, which involves placing a perforated membrane in the furrow between two cultivation ridges and covering it with a plastic film, water can flow between the plastic film and the perforated membrane and infiltrate into the soil through pores on the membrane under non-pressure gravity irrigation. This technique not only saves irrigation equipment costs, but it also eradicates blocking, and has better irrigation uniformity [12].

To date, numerous studies have explored the effects of fertilizer application rate on crop growth, yield, and quality [4, 9, 13]. For example, Qu et al. [14] found that the yield increased with a rising fertilizer application rate up to a point, after which yield decreased in cucumbers grown in substrate bags in spring. Zhang et al. [15] noticed that, compared to the conventional fertilizer application method, a controlled-release fertilizer management method significantly increased yield, with more accumulated total dry weight in bitter gourd. Currently, the techniques integrating microporous membrane water and fertilizer application are largely based on drip irrigation tapes, and the possibility of non-pressure gravity irrigation by water-fertilizer integration using microporous membranes has rarely been explored.

In this research, we employed the integrated microporous membrane water and fertilizer technique for non-pressure gravity irrigation, to determine the optimal fertilizer application rate of spring tomato cultivated in a plastic greenhouse under a specific irrigation level and to clarify the fertilizer requirements of tomato, to thus provide a theoretical basis for efficient fertilizer application and cultivation.

## Material and methods

### Experimental material, site, and time

The ‘K1602’ tomato variety was employed in this study. Water-soluble fertilizer (N:P_2_O_5_:K_2_O, 18:7:20) and base fertilizer (N:P_2_O_5_:K_2_O, 15:15:15) were obtained from Yichuan Fufeng Plant Nutrients & Fertilizers Co., Ltd. The perforated plastic film was 60 cm wide and 6.4 m long. Holes (3-mm diameter) in the film were spaced at intervals of 20 cm lengthwise and 12.5 cm across, with three holes punched in each row. The experiment was performed at Zhengzhou Zhengyan International Seed Technology Exhibition Park (Xinzheng, China) (34°16′ to 34°39′ N, 113°30′ to 113°54′ E), from March 16 to July 12, 2017.

### Experimental design and treatment

Four different fertilizer levels were examined in this experiment: 1290 kg/ha (FA), 1140 kg/ha (FB), 990 kg/ha (FC), and 840 kg/ha (FD), respectively. The local traditional fertilizer application level (1875 kg/ha) was set as the control (CK). The irrigation amount and target yield of all treatments were 1650 m^3^/ha and 11.25 t/ha, respectively.

Tomato seedlings were planted at the six-leaf stage. Each plot area was 7.8 m^2^ (6 m × 1.3 m), with three biological replicates. The base fertilizer was applied in the form of dried chicken manure (1.5 × 10^4^ kg/ha) and compound fertilizer (N:P_2_O_5_:K_2_O, 15:15:15, 525 kg/ha). The CK treatment used furrow irrigation to fertilize plants, and integrated irrigation and fertilizer application was performed in other treatments. The times and amounts of irrigation and fertilization are listed in Table 1.

**Table 1:** Irrigation and fertilization regime after tomato planting.

Days after planting (d)		14	50	59	70	78	84	90	96	102	108	115	126
Irrigation amount (ton/ha)		90	180	180	180	180	180	180	180	180	180	180	180
Topdressing amount (kg/ha)	FA	0	180	180	180	180	180	180	180	120	90	0	0
	FB	0	120	150	180	180	120	120	90	90	90	0	0
	FC	0	90	150	180	180	120	120	60	60	60	0	0
	FD	0	90	120	150	90	90	90	60	60	60	0	0
	CK	Twice in April, 150 kg/time^/^ha. Three times in May, 225 kg/time^/^ha. Three times in June, 300 kg/time^/^ha.											

### Measurements of crop parameters

For analysis, seven plants were randomly selected from each treatment from one replicate. Plant height, stem diameter, and leaf number were measured 20, 40, and 60 d after planting. Fresh and dry weight as well as the nitrogen (N), phosphorus (P), and potassium (K) contents of plants were measured after uprooting. Soil samples before transplanting and after uprooting were collected from the 0–20, 20–40, and 40–60 cm soil layers using a five-point sampling method [16]. The third truss fruits were picked to determine fruit quality.

Plant height was measured using a tape measure, while basal stem diameter (at the midpoint between the stem base and cotyledon) was measured with Vernier calipers. After fresh weights of roots, stems, and leaves were measured, samples were dried at 105°C for 15 min, and then dried at 85°C until reaching a constant mass. Leaf number was counted on plants (those less than 5 cm in length were excluded). The N, P, and K contents were measured using the Kjeldahl method, vanado-molybdate colorimetry, and flame photometry, respectively [17, 18]. The contents of soluble sugars, soluble proteins, vitamin C, soluble solids, and organic acids were measured according to the methods of Rahi et al. [19] and Tudor-Radu et al. [20]. The lycopene content was calculated according to the method of Kumar et al. [21], while the fruit yield was measured for each plot. Partial factor of productivity (PFP) was calculated as

PFP=Y/I (1)

where *Y* (kg/ha) is total yield of crop fruit and *I* (kg/ha) is the total fertilizer application amount throughout the growth period.

### Data analysis

All data are presented as the mean ± standard error (SE) of three replicates and were analyzed using Data Processing Software (DPS, version 7.05) following one-way analysis of variance (ANOVA). Significant differences (*P* < 0.05) among treatment means after controlling for multiple comparisons were determined from a least significant difference (LSD) test.

## Results

### Effects of different fertilizer application rates on plant growth at different periods

Fertilizer application rate affected plant growth (Table 2). At the 20^th^ d after planting, plant height and stem diameter of FB and FC plants and leaf number of FA and FC plants, were significantly higher than those of CK plants. By the 40^th^ d, plant height of FA and FB plants, stem diameter of FC plants, and leaf number of FA plants were respectively increased by 17.70%, 17.04%, 15.54%, and 8.14%, compared to CK plants. By the 60^th^ d, there were no significant differences among various fertilizer treatments in plant height, stem diameter, or leaf number. These results indicated that fertilizer mainly had an effect during the early and intermediate stages of plant growth, among which the effect of the FC treatment was best.

**Table 2:** The height, stem diameter, and leaf number of tomato plants grown under different fertilization rates at different periods.

Fertilizer treatment	Plant height (cm)			Stem diameter (mm)			Leaf number		
	20 d	40 d	60 d	20 d	40 d	60 d	20 d	40 d	60 d
FA	51.7±0.9b	88.7±3.5a	127.0±1.7a	4.65±0.19ab	11.91±0.35b	13.39±0.40a	7.9±0.3ab	11.4±0.4a	18.3±0.8a
FB	58.7±0.6a	88.2±2.6a	128.4±0.8a	5.11±0.18a	12.49±0.58ab	13.44±0.43a	7.3±0.2abc	10.4±0.2b	19.1±0.4a
FC	58.4±1.3a	80.1±6.8ab	125.6±2.6a	5.07±0.21a	13.16±0.35a	14.27±0.29a	8.0±0.3a	11.1±0.3ab	18.3±0.8a
FD	53.0±0.9b	63.9±1.0c	123.3±2.0a	4.55±0.12ab	11.63±0.18b	13.84±0.12a	7.1±0.3bc	10.4±0.2b	18.7±0.5a
CK	51.4±1.3b	75.4±1.0b	122.4±2.5a	4.48±0.23b	11.39±0.36b	13.04±0.60a	6.9±0.36c	10.6±0.2b	19.3±0.4a

Data are means ±SE. Different letters within the same column denote statistically significant differences between treatments (P<0.05). The same below.

### Effects of different fertilizer application rates on fresh and dry weight

Fresh and dry weight of roots under FA treatment were highest, which were 37.31% and 47.00% higher than those under the CK treatment, respectively (Table 3). The fresh weight of stems was not significantly different among the FB, FC, and CK treatments, but significantly greater than that in the FD treatment. The dry weight of stems was not significantly different among the FB, FC, FD, and CK treatments, which were each significantly lower than that in the FA treatment. Fresh and dry weights of leaves increased as fertilizer application amount decreased except for the FD treatment; those of the FA, FB, and FC treatments were significantly higher than those in the CK treatment, with the FC treatment having the highest values, 97.37% and 95.69% higher than those in the CK treatment, respectively.

**Table 3:** Fresh and dry weight of tomato plants grown under different fertilizer application rates.

Fertilizer treatment	Fresh weight (g)			Dry weight (g)		
	Root	Stem	Leaf	Root	Stem	Leaf
FA	51.00±2.37a	356.86±11.10b	533.57±21.07b	11.51±0.70a	64.86±2.79a	67.93±3.52c
FB	48.43±3.12ab	389.14±7.35a	581.29±18.68ab	9.36±0.27b	51.57±1.56bc	76.35±2.43b
FC	44.00±1.23b	372.57±11.58ab	600.00±26.54a	8.78±0.27bc	55.41±1.43b	92.62±2.39a
FD	32.71±0.68c	310.71±8.72c	332.29±6.09c	6.90±0.14d	47.85±1.25c	49.60±1.29d
CK	37.14±0.67c	367.29±5.40ab	304.00±5.40c	7.83±0.13cd	50.30±1.92bc	47.33±0.99d

### Effects of different fertilizer application rates on N, P, and K contents of tomato plants

Total N content in the roots was not significantly different among the FC, FD, and CK treatments and significantly greater than that in the FA and FB treatments (Table 4). Total N content in the stems and leaves were lowest under the FD and FC treatments, respectively, which were not significantly different compared with the CK treatment. The P content of the roots under the FC treatment was higher than that in the CK treatment; no significant differences in P content of the roots or stems were recorded between any other fertilizer treatment and the CK treatment. P content of the leaves under the FA treatment was higher than in the CK treatment, while that in the other fertilizer application treatments was lower than that in the CK treatment. The K content of the roots and stems were greatest under the FD and FC treatments, respectively, and significantly greater than that in the CK treatment. However, the K content of leaves in the FC and FD treatments were lower than in the CK treatment.

**Table 4:** N, P and K content of tomato plants grown under different fertilizer application rates.

Fertilizer treatment	N content (%)			P content (%)			K content (%)		
	Root	Stem	Leaf	Root	Stem	Leaf	Root	Stem	Leaf
FA	0.18±0.00c	0.37±0.01bc	0.50±0.01b	0.11±0.01c	0.25±0.00a	0.40±0.00a	0.84±0.01d	0.72±0.02d	0.93±0.03a
FB	0.19±0.00b	0.44±0.02a	0.56±0.00a	0.12±0.00c	0.24±0.00ab	0.31±0.01c	0.84±0.01d	0.35±0.02e	1.00±0.02a
FC	0.24±0.00a	0.41±0.01ab	0.46±0.01c	0.15±0.01a	0.25±0.01a	0.26±0.01d	0.90±0.02c	1.36±0.04a	0.80±0.01b
FD	0.23±0.00a	0.33±0.01d	0.50±0.01b	0.14±0.00ab	0.22±0.00b	0.19±0.01e	1.06±0.02a	1.14±0.01b	0.80±0.03b
CK	0.24±0.00a	0.36±0.01cd	0.46±0.01c	0.13±0.00bc	0.24±0.01ab	0.36±0.01b	0.97±0.01b	0.94±0.01c	0.96±0.01a

### Effects of different fertilizer application rates on N, P, and K contents in different soil layers

To analyze the nutrient surpluses in the soil, the N, P, and K contents of soil before planting and after uprooting in different layers were measured (Table 5). The N content of the CK treatment in the top soil layer (0–20 cm) was not significantly different from the soil before transplanting, while the same layers of FA and CK soil were greatly higher in N content than those of the FC and FD treatments. The P content of the soil before transplanting was sharply lower than the FA and FC soil, and that no significant differences were found among the FB, FD, and CK treatments. For K content, there were no significant differences among fertilizer levels, and that of each treatment was significantly lower than in the soil before transplanting. In the 20–40 cm soil layer, the N contents of the CK treatment was remarkably greater than in the soil before transplanting and other fertilizer treatments. The P content in CK soil was significantly lower than that in the soil before transplanting and FA soil and sharply higher than in the soil in the other treatments. Except for the FB treatment soil, the K content of CK soil was not notably different from the other treatments. In the drip soil layer (40–60 cm), the N content of FC and FD soils were significantly lower than that of CK soil. The P content of CK soil was highest, while that of FC soil was lowest. The K content of CK soil was significantly lower than of FA and FB soils and not different from that of basic soil or the other fertilizer levels. Thus, N, P, and K enrichment under the FC and FD treatment was decreased in drip soil.

**Table 5:** N, P, and K contents of different soil layers under different fertilizer application rates.

Fertilizer treatment	N content (%)			P content (%)			K content (%)		
	0–20 cm	20–40 cm	40–60 cm	0–20 cm	20–40 cm	40–60 cm	0–20 cm	20–40 cm	40–60 cm
No fertilizer	0.19±0.00a	0.14±0.00e	0.09±0.00d	0.05±0.00cd	0.20±0.01a	0.06±0.00b	0.41±0.02a	0.42±0.01a	0.38±0.01bc
FA	0.20±0.01a	0.17±0.00c	0.15±0.00b	0.36±0.01a	0.21±0.01a	0.07±0.01b	0.37±0.03b	0.41±0.01a	0.48±0.01a
FB	0.16±0.00c	0.15±0.00d	0.17±0.00a	0.05±0.00cd	0.04±0.00c	0.07±0.00b	0.35±0.01b	0.39±0.01a	0.42±0.01b
FC	0.18±0.00b	0.18±0.00b	0.13±0.00c	0.23±0.01b	0.03±0.00c	0.04±0.00c	0.34±0.01b	0.36±0.01b	0.36±0.01c
FD	0.15±0.00c	0.17±0.00c	0.13±0.00c	0.04±0.00d	0.03±0.00c	0.05±0.00c	0.34±0.01b	0.41±0.01a	0.40±0.02bc
CK	0.19±0.00a	0.20±0.00a	0.15±0.00b	0.07±0.00c	0.09±0.00b	0.09±0.00a	0.34±0.01b	0.39±0.01a	0.39±0.01bc

### Effects of different fertilizer application rates on tomato quality

Soluble sugar content under the FD treatment was highest, 2.4% higher than that under the FC and CK treatments (Table 6). There was no significant difference in organic acid content under the CK, FC, and FD treatments. The sugar-acid ratio of under the FA, FB, and FD treatments were markedly greater than that of the CK treatment (16.57%, 17.71%, and 11.14% higher, respectively), while those of the FD and FC treatments were not notably different. The lycopene content under the FB, FC, and FD treatments were all significantly higher than that under the CK treatment, with that of the FC treatment being the highest, 39.58% higher than that of the CK treatment. The soluble protein content of the CK treatment was significantly lower than that of the FA treatment (by 8.19%), but greater than that of the other fertilizer treatments. The soluble solid content under the CK treatment was highest, but not significantly different than that under the FA, FC, and FD treatments. The vitamin C content under the FA treatment was greatest, but that was not distinctly different from that of the CK treatment. On the whole, fertilizer treatments FC and FD were more effective in improving tomato fruit quality.

**Table 6:** Fruit quality of tomato grown under different fertilizer application rates.

Fertilizer treatment	Soluble sugar	Organic acid	Sugar-acid ratio	Lycopene	Soluble protein	Soluble solid	Vitamin C
	(g/100 g)	(%)	(%)	(mg/L)	(mg/g)	(%)	(mg/100 g)
FA	0.40±0.00c	5.07±0.20bc	7.95±0.23a	0.50±0.00e	3.04±0.05a	0.57±0.02ab	13.83±0.30b
FB	0.40±0.00c	4.98±0.14c	8.03±0.21a	0.60±0.00b	1.71±0.04c	0.53±0.02b	12.41±0.29c
FC	0.41±0.00b	5.55±0.12ab	7.41±0.16ab	0.76±0.00a	1.00±0.01d	0.57±0.02ab	15.00±0.22a
FD	0.42±0.00a	5.55±0.19ab	7.58±0.28a	0.57±0.00c	1.10±0.04d	0.59±0.01ab	11.17±0.26d
CK	0.41±0.00b	6.03±0.15a	6.82±0.17b	0.55±0.00d	2.81±0.04b	0.60±0.02a	14.27±0.18ab

### Effects of different fertilizer application rates on yield and PFP

The FA yield per plant was highest, being significantly higher than that of the CK treatment (by 8.82%), while those of the other fertilizer treatments were not significantly different from that of the CK treatment (Table 7). Plot yield for the FA and FB treatments were significantly higher than that under the CK treatment (5.65% and 6.89%, respectively). PFP under the four fertilizer levels were significantly (39.71%, 54.08%, 47.83%, and 75.72%, respectively) greater than that under the CK treatment, while the FD treatment had the highest PFP, with a plot yield that was not significantly different from that of the CK treatment.

**Table 7:** Yield and PFP of tomato grown under different fertilizer application rates.

Fertilizer treatment	Yield per plant (kg)	Plot yield (kg)	PFP (kg/kg)
FA	3.95±0.09a	107.21±1.19a	69.62±0.77d
FB	3.73±0.03ab	108.46±1.63a	76.78±1.15b
FC	3.40±0.11c	94.41±0.83c	73.44±0.64c
FD	3.70±0.05ab	101.41±1.37b	87.56±1.18a
CK	3.63±0.14bc	101.47±1.00b	49.83±0.49e

## Discussion

Compared to the use of furrow irrigation to apply fertilizer, the microporous membrane water-fertilizer integration technique (which functions like drip irrigation technology) was able to reduce topdressing amounts and promote normal growth in tomato plants. The drip irrigation equipment needs a large initial investment, however, the microporous membrane used in this study was made from a used shed film, which is more economic for growers than installing a drip irrigation system.

Scientific fertilizer application offers an important means of improving crop growth, protecting the ecological environment, and maintaining agricultural sustainability. Plant fresh and dry weight, which reflect the accumulation of plant biomass to some extent, constitute important indicators of growth vigor [15, 22]. In general in this study, under the same irrigation conditions, plant growth in the early and intermediate stages increased with decreasing fertilizer application up to a point, after which they decreased. Fertilizer application improved availability of NPK in root zone, leading to an increase in uptake of nutrients to the plant. Many studies have demonstrated this increment in nutrients contributed to plant biomass accumulation is due to higher leaf photosynthetic capacity [1, 23]. However, excess fertilizer application would result in low water availability to plant due to high osmotic conditions in soil, and therefore stunted plants [24]. Our results also showed that the total NPK contents of FC plants were the highest, corresponding to the best plant growth and highest dry weight.

Nutrient content in the surface soil is influenced by fertilizer application rate, irrigation, and plant species, as nutrients are partially taken up by plants and partially migrate downward deep into soil as moisture moves [25, 26]. In the present study, N and K were enriched in deep soil, especially under high fertilizer application, but the contents of N and K in 0–20 cm soil were similar or lower than that in soil before treatment. In soil, N and K are mobile nutrients. Displacement of N and K through the soil profile often occur with irrigation during over application [27, 28]. This may be responsible for the N and K distribution in soil under high fertilizer application. On the contrary, P enrichment was found in topsoil, which may be due to high Ca^2+^ concentrations in water and soil in experimental site [29, 30]. In calcareous soils, P can be immobilized into insoluble compound, such as calcium-phosphate minerals, leading to low mobility [31, 32]. The high residues of nutrient would increase the risk of groundwater pollution. Thus, the 840 kg/ha fertilizer application rate met the nutrient needs of tomato plants, without leaving excess residues in the soil, making it the optimal fertilizer application rate.

In this study, the PFP under four fertilization levels were significantly higher than that of the CK treatment. This suggested that the microporous membrane water-fertilizer integration technique could improve PFP. Moreover, PFP is greatly affected by fertilizer supply level. We found that the highest PFP occurred under the lowest fertilizer amount (840 kg/ha). Previous studies also confirmed that the highest PFP often occur under a low fertilizer supply level [26, 33]. According to fertilizer response function, positive effects of fertilizer on yield may be responsible for the high PFP under low fertilizer supply level [34]. On the other hand, high fertilizer input increased nutrient leaching losses, and therefore low PFP [22, 35].

Fertilizer is an important determinant for yield formation. High fertilizer application under non-pressure gravity irrigation enhanced yield compared with CK while no significant difference in yield was observed between lowest fertilizer treatment (840 kg/ha) and CK. Interestingly, the yield was lowest under 990 kg/ha treatment. This may be attributed to more photosynthate allocation to vegetative growth, thereby impacting their reproduction. Under non-pressure gravity irrigation, the fertilizer application rate was declined by 31.2%-53.6% compared with CK. Thus, the decreased fertilizer supply under non-pressure gravity irrigation have no negative effect on tomato plant growth and yield. Specifically, the 840 kg/ha fertilizer treatment achieved the highest economic value under non-pressure gravity irrigation. Although the per plant and plot yields under 840 kg/ha fertilizer treatment were 8.8% and 5.7% lower than that under the 1290 kg/ha fertilizer treatment, the fertilizer input decreased by 34.8%. Considering the fertilizer input, nutrient residues and yield, 840 kg/ha fertilizer application is recommended.

## Conclusion

In conclusion, the 840 kg/ha fertilizer application rate most effectively improved the yield, PFP, and soil productivity maintenance, with better quality of tomato fruits. Our results preliminarily identified a specific irrigation and fertilization regime for tomato cultivation under non-pressure gravity irrigation. In future work, the optimal application proportion of NPK for tomato should be assessed under non-pressure gravity irrigation.

## Supporting information

S1 Data (XLSX)
